# Examining the Use of Polyphenols and Sugars for Authenticating Honey on the U.S. Market: A Comprehensive Review

**DOI:** 10.3390/molecules29204940

**Published:** 2024-10-18

**Authors:** Kate Nyarko, Stephen Mensah, C. Michael Greenlief

**Affiliations:** Department of Chemistry, University of Missouri, Columbia, MO 65211, USA; knzhb@missouri.edu (K.N.); smfrb@missouri.edu (S.M.)

**Keywords:** honey, authenticity, polyphenol markers, sugar markers, United States, honey origin

## Abstract

The rise in honey production and imports into the United States necessitates the need for robust methods to authenticate honey origin and ensure consumer safety. This review addresses the scope of honey authentication, with a specific focus on the exploration of polyphenols and sugar markers to evaluate honeys in the U.S. In the absence of comprehensive federal standards for honey in the United States, challenges related to authenticity and adulteration persist. Examining the global landscape of honey authentication research, we observed a significant gap in the literature pertaining to U.S. honeys. While honeys from Europe, Australia, New Zealand, and Asia have been extensively studied, the decentralized nature of the U.S. honey market and the lack of comprehensive standards have limited the number of investigations conducted. This review consolidates the findings of global honey studies and emphasizes the need for further research studies on honey authenticity markers within the United States. We also explore previous studies on the U.S. that focused on identifying potential markers for honey authenticity. However, the inherent variability in polyphenol profiles and the lack of extensive studies of the sugar contents of honey on a global scale pose challenges to establishing universal markers. We conclude that by addressing these challenges, the field of research on polyphenols and sugars in honey can move toward more reliable and standardized methods. This advancement will enhance the use of polyphenols and other constituents like sugars as authenticity markers, ultimately benefiting both researchers and the honey industry in ensuring honey quality.

## 1. Introduction

The U.S. honey market faces several significant challenges that threaten both the industry and consumer trust. One of the most pressing issues is the growing prevalence of honey adulteration, where products labeled as “honey” are mixed with cheaper sweeteners, like corn syrup or rice syrup [1]. This practice not only deceives consumers but also undermines the value of pure honey. The influx of imported honey, some of which is mislabeled or adulterated, further complicates the issue. With the U.S. being a major honey importer, the lack of stringent regulations and the difficulty in detecting adulteration pose a serious threat to the market’s integrity. Amid these challenges, the accurate determination of the constituents of honey polyphenols and sugars may be crucial for identifying authentic honey. Polyphenols and sugars, such as glucose and fructose, are natural compounds found in plants, and they are present in honey as a result of bees collecting nectar from various floral sources. These compounds are not only beneficial to human health due to their antioxidant properties and health-promoting properties [2,3], but they also serve as potential markers for honey authenticity. Each type of honey has a unique polyphenol profile that reflects its floral origin, making these fingerprints valuable tools for verifying the authenticity of honey. Honey adulteration poses significant challenges for beekeepers, the honey industry, and researchers alike. The intentional mixing of lower-quality substances with pure honey undermines product authenticity, leading to unfair competition and reduced market prices for genuine honey producers. This economic disparity highlights the urgent need to verify the geographical origin of honey, as mislabeling remains a prevalent issue [4,5]. Knowing the origin and production source of honey is essential for assessing its quality, and there is extensive literature on honey authentication based on geographical origin. Many studies have focused on honey from international markets, including those in Europe, Asia, and Africa. Current methods for identifying the geographic and botanical origins of honey rely primarily on fingerprinting techniques combined with chemometrics and specific markers, such as sugars, pollen, phytochemicals, and biomacromolecules (e.g., proteins and DNA). Wang et al. [6] systematically reviewed these markers, demonstrating their ability to differentiate honeys based on their origins. The study also highlighted the need for simpler and more sensitive approaches. Enhancing current methods by integrating multiple parameters and employing advanced statistical analyses is essential for improving the accuracy of honey authentication.

For instance, spectroscopic techniques, such as NMR and Raman spectroscopy, coupled with multivariate statistical analyses, have effectively identified Romanian honey by its geographical origin [7]. These methods have successfully identified various honey types, including Acacia, linden, sunflower, and polyfloral honeys. Similarly, multivariate data analyses and machine learning have demonstrated their effectiveness for distinguishing between honey samples based on geographical origin by analyzing diverse descriptive parameters, such as their atomic spectra, physicochemical traits, and sensory data, achieving high accuracy in exploratory and predictive analyses [8]. Some researchers have also identified volatile compounds in honey as potential markers for determining its geographical origin. For example, Kaskoniene and colleagues [9] utilized SPME-GC-MS and chemometric tools to differentiate multifloral honeys from various regions in Lithuania by detecting different classes of chemical compounds, including alcohols, ketones, aldehydes, acids, terpenes, and hydrocarbons. Additionally, the volatile profiles of Sudanese honeys have revealed various chemical groups [10]. Aliferis and co-workers applied a similar method to distinguish between citrus and thyme honeys from different locations in Greece [11].

Detecting honey adulteration involves analyzing sugar profiles, particularly the presence of oligosaccharides that do not naturally occur in honey. High-fructose corn syrup (HFCS) and other syrups can be identified as adulterants, even at low concentrations [12,13,14]. By integrating chemometric and pattern recognition tools, such as principal component analysis (PCA), partial least squares–linear discriminant analysis (PLS-LDA), and multiple linear regression (MLR) with voltametric electronic tongues, authentic honey samples from Romania were differentiated from those adulterated with sugars like fructose and malt wort [15,16]. This approach achieved an impressive 83.33% correct classification of authentic versus adulterated honey using the LDA model on electronic tongue data. Additionally, some studies have employed δ13C isotope ratio methods to identify honey adulteration, particularly when bees are fed significant volumes of commercial sugar syrups [17].

Owing to the successful integration of honey composition analysis with chemometric tools, researchers have increasingly focused on exploring honey’s complex composition, with a specific focus on its sugars, phenolics, volatiles, and other constituents, by utilizing their unique fingerprints to distinguish authentic honeys from adulterated ones [18,19,20,21]. Despite the promise of these constituents, including polyphenols and sugars, as authenticity markers, research in this area faces its own set of challenges. For instance, the variability in polyphenol content across different honey samples makes it difficult to establish consistent markers. Factors, such as climate, soil composition, and even the bee species, can influence these profiles, adding additional complexity to the research [22]. Additionally, the lack of standardized methods for polyphenol extraction and analysis hinders the comparability of results across different studies and laboratories [23]. This inconsistency makes it challenging to develop universally accepted authenticity markers for honey products [10]. Despite the aforementioned challenges, polyphenols along with sugars are critical tools for honey authentication. While polyphenols offer unique insights into the floral and geographical origins, sugars—particularly their composition and ratios—are equally valuable for distinguishing adulterated from authentic honey.

**Figure 1:**
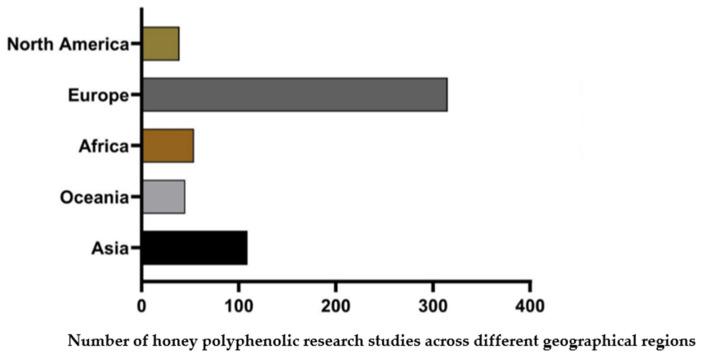
A graph showing the total number of honey polyphenol research papers published across different geographical regions during the years 1990–2024.

To the best of our knowledge, not much work has been performed to establish the polyphenol profiles and sugar content of honey from North America, despite the significant contribution of honey to the agricultural sector in countries like the United States. Many of the studies and reviews from 1990 to 2024 surveyed in this study focused on honeys from different parts of the world. Most of these reviews were found with keywords, such as “honey authenticity”, “honey adulteration”, “honey floral and geographical markers”, “honey polyphenols”, “honey sugars”, “honey origin”, “U.S. honeys”, and “honey authenticity markers”, from Scopus, Google Scholar, Web of Science, MDPI, ScienceDirect, PLOS, Taylor and Francis, and PubMed Central. Our online searches found that the authenticity of honey from the regions of Europe, Australia and New Zealand, the Middle East, and Asia were the most studied over the last two decades, as shown in Figure 1.

**Figure 2:**
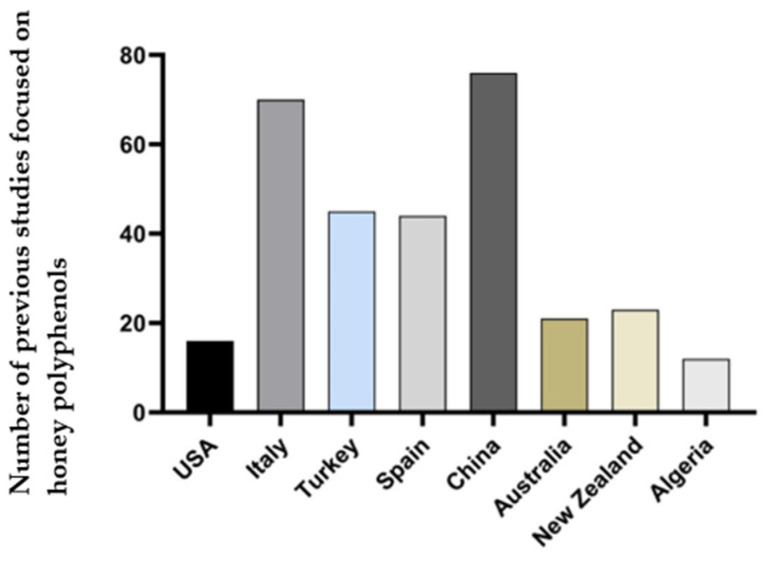
A summary of the number of studies (1990–2024) focused on honey phenolic research from several honey-producing countries around the world and the U.S.

The studies on honey from countries including New Zealand, China, Italy, Spain, Poland, and Turkey, presented in Figure 2, have gained much attention due to their distinctive characteristics and the prevalence of specific concerns, such as their adulteration with foreign additives like sugars. Additionally, the diverse geography of the United States makes it challenging to generalize findings about the entire U.S. honey market. This decentralized structure has, in a sense, shielded U.S. honey from the intense study that other regions have undergone. The lack of comprehensive federal standards for honey in the United States has further resulted in potential issues related to honey authenticity and adulteration. In a recent study, Lawag et al. [24] found that within the Americas, countries such as Brazil, Argentina, Chile, and Cuba have been the central focus in terms of honey phenolics research. In addition, they highlighted that China stands out as the global leader in this research, contributing 76 reports on the phenolic constituents of monofloral honeys, with 76% of these studies focusing on locally sourced varieties. Italy follows closely with 74 papers, while Turkey has also actively engaged in this research, with 45 reports exclusively on Turkish honeys [24].

This review offers a comprehensive analysis of the challenges to honey authentication, highlighting the potential of using honey components, including polyphenols and sugars, as effective tools for authenticating honey in the U.S.

## 2. Chemical Composition of Honey

### 2.1. Polyphenol Composition of Honey

Honey contains a significant class of compounds known as polyphenols, which are secondary metabolites that are widespread in the plant kingdom. The structural framework of polyphenols comprises aromatic rings bonded to phenol functional groups. This diverse class of compounds can be categorized into distinct groups, primarily phenolic acids, flavonoids, tannins, lignans, coumarins, and stilbenes [25]. Among these, flavonoids and phenolic acids stand out due to their abundance in various foods, with well-documented dietary importance. Polyphenols, particularly flavonoids and phenolic acids, have been the subject of extensive research, highlighting their biological activities and significance in plant studies. Most polyphenolic compounds exhibit either aromatic or aliphatic structures fused with phenol groups. Phenolic acids, characterized by the presence of a hydroxyl functional group in their structure, are subdivided into hydroxybenzoic acids (C6-C1) and hydroxycinnamic acids (C6-C3) [26]. Variations in these structures are determined by the degree of methylation and hydroxylation [27], contributing to the diversity of phenolic compounds found in honey and other natural sources.

In contrast, flavonoids exhibit a distinctive structure characterized by two benzene rings with a central pyran ring, forming a general structure of C6-C3-C6. Flavonoids can be categorized into distinct groups based on the position of the central pyran ring to which the B ring is attached, and the degree of unsaturation of the C ring [28]. The extensive distribution and study of flavonoids in plants has led to the identification of subgroups, such as flavanones, isoflavones, flavones, flavonols, flavanols, anthocyanins, and catechins. Flavonoids and phenolic acids are among the most thoroughly investigated phenolic compounds in plants, with numerous subgroups present in a variety of plant species. In terms of honey, these compounds have also been widely explored across different botanical and geographical origins [29,30,31,32,33]. Common examples of phenolic compounds identified in honey from different floral and geographical sources include ferulic acid, caffeic acid, apigenin, pinocembrin, hydroxybenzoic acid, gallic acid, kaempferol, myricetin, naringenin, and quercetin. Table 1 provides a comprehensive list of the common phenolic compounds that have been identified in monofloral honeys from diverse geographical sources.

**Table 1:** Comprehensive list of common flavonoids and phenolic acids identified in monofloral honeys [34,35].

Flavonoids in Honey	Chemical Formula	Botanical Sources	Flavonoid Class
Kaempferol	C_15_H_10_O_6_	Buckwheat, acacia, citrus, chestnut, ginger, heather, honeydew, linden, litchi	flavonol
Galangin	C_15_H_10_O_5_	Acacia, manuka, rosemary, sage, sunflower, lotus, linden, honeydew, heather, eucalyptus, citrus, buckwheat, chestnut	flavone
Myricetin	C_15_H_10_O_8_	Linden, lotus, pine, spruce, sunflower, thyme, honeydew, heather, gelam, fir, eucalyptus, citrus, chestnut, buckwheat, strawberry tree, tualang	flavone
8-methoxykaempferol	C_16_H_12_O_7_	Manuka, rosemary	flavonol
Myricetin-3-methylether	C_16_H_11_O_8_	Australian jelly bush, heather, manuka	flavonol
Quercetin	C_15_H_10_O_7_	Australian jelly bush, chestnut, citrus, clover, eucalyptus, gelam, ginger, heather, linden, Japanese grape, mastic, pine, manuka, acacia, rosemary	flavonol
Isorhamnetin	C_16_H_12_O_7_	Acacia, citrus, eucalyptus, lotus, linden, honeydew, *diplotaxis tenuifolia*	flavonol
Genistein	C_15_H_10_O_5_	Acacia, clover, cedrus	isoflavone
Anthocyanidins	C_15_H_11_O^+^	Acacia, manuka, tualang	
Hesperetin	C_16_H_14_O_6_	Citrus, eucalyptus, gelam, lotus	flavanone
Pinocembrin	C_15_H_12_O_4_	Spruce, lotus, manuka, rosemary, strawberry tree, sunflower, linden, honeydew, fir, eucalyptus, citrus, chestnut, acacia	flavanone
Naringenin	C_15_H_12_O_5_	Lavender, lemon, linden, orange, rosemary, spruce, tualang, fir, honeydew, *Rhododendron*	flavanone
Acacetin	C_16_H_12_O_5_	Acacia, *Robina Pseudoacacia*	flavone
Pinobanksin	C_15_H_12_O_5_	Sunflower, strawberry tree, spruce, rosemary, manuka, lotus, linden, leatherwood, fir, honeydew, eucalyptus, citrus, chestnut	flavanone
(−)-Catechin	C_15_H_14_O_6_	Heather, tualang	catechins
(−)-Epicatechin		Lavender, litchi, oak	catechins
Chrysin	C_15_H_10_O_4_	Citrus, heather, chestnut, honeydew, acacia, linden, rosemary, buckwheat, sunflower, thyme, pine	flavone
Apigenin	C_15_H_10_O_5_	Citrus, fir, honeydew, eucalyptus, chestnut, acacia, *Astralagus* spp.	flavone
Luteolin	C_15_H_10_O_6_	Honeydew, lavender, linden, manuka, chestnut, thyme, sage, sunflower, lotus, eucalyptus	flavone
Ellagic acid	C_14_H_6_O_8_	Buckwheat, gelam, heather, manuka, Australian jelly bush	Tannins
**Phenolic Acids**			
Ferulic acid	C_10_H_10_O_4_	Chaste tree, chestnut, citrus, eucalyptus, canola, buckwheat, heather, jujube	
Caffeic acid	C_15_H_10_O_6_	Chestnut, citrus, clover, chaste tree, linden, lotus, gelam, eucalyptus, manuka, rosemary, sunflower, thyme, tualang	
Homogentisic acid	C_8_H_8_O_4_	Strawberry tree, chaste tree, thyme	
Gentisic acid	C_7_H_6_O_4_	Eucalyptus, pine, thyme, Cedrus, carob (*Ceratonia silique*)	
3-phenyllactic acid	C_9_H_10_O_3_	Leatherwood, mauka, milk thistle, Kamahi, chestnut	
*p*-coumaric acid Cinnamic acid	C_9_H_8_O_3_ C_9_H_8_O_2_	Acacia, *astralagus* spp., *Azadirachta indica*, buckwheat Plum, willow, lime, rapeseed	
Gallic acid	C_7_H_6_O_5_	Japanese grape, Litchi, Kamahi, honeydew, gelam, chestnut, Australian jelly bush, acacia, linden, heather, ginger	
Chlorogenic acid	C_16_H_18_O_9_	Heather, honeydew, thyme, acacia, buckwheat	
Rosmarinic acid	C_18_H_16_O_8_	Manuka, mint, heather, buckwheat	
Syringic acid	C_9_H_10_O_5_	Lavender, leatherwood, lotus, fir, citrus, pine, oak, thyme, tualang, ginger, heather	
Benzoic acid	C_7_H_6_O_2_	Manuka, citrus, heather, tualang, acacia, lotus, eucalyptus	
4-hydroxybenzoic acid	C_7_H_7_O_3_	Chaste tree, buckwheat, acacia, chestnut, clover, heather, linden, lotus	
Vanillic acid	C_8_H_8_O_4_	Acacia, buckwheat, Cedrus, chestnut, eucalyptus, heather, linden, citrus	

### 2.2. The Sugar Composition of Honey

The detection of honey adulteration is usually difficult, owing to the variations in its composition. The sugar composition, however, is largely dependent on the botanical origin or the types of flower nectar used by the bees, and the geographical origin, climate, processing, and storage of the honey [36]. Several studies have been carried out globally to identify the sugars present in honey. These sugars include fructose, glucose, sucrose, rhamnose, trehalose, nigerobiose, isomaltose, maltose, maltotetraose, maltotriose, maltulose, melezitose, melibiose, nigerose, palatinose, raffinose, erlose, and others [37]. Over 22 different sugars have been identified in honey. Among these, fructose and glucose are the most abundant [38]. In honey, about 70–80% *w*/*w* of its constituents are carbohydrates and 10–20% *w*/*w* are water [39]. The amount of carbohydrates in honey by dry weight, occurring mainly as sugars, has been reported to be approximately 95% *w*/*w*. This includes 65–80% glucose and fructose. The amount of fructose in honey is more than glucose, and accounts for the extreme sweetness of honey. The concentrations of the different sugars in honey in terms of their percentages have been reported to be about 38.19% fructose, 31.28% glucose, 7.97% maltose, 4.5% sucrose, and about 0.86% higher sugars [40,41].

Various factors can contribute to variations in honey constituent levels. However, key indicators, such as the combined levels of fructose and glucose, the fructose/glucose ratio (F/G), and the ratio of glucose to water (G/W) play crucial roles in assessing the quality of honey [42,43]. The F/G ratio is a determinant of the ability of honey to crystallize, although the G/W ratio may also be used. Accordingly, the F/G ratio is helpful for the determination of honey crystal size [36]. It is estimated that honey samples with F/G ratios exceeding 1.33 are less prone to crystallization, whereas those with F/G ratios below 1.11 tend to crystallize quickly [44,45]. These ratios are important factors for honey quality. It is expected that the percentage sucrose in honey does not exceed 5%. Therefore, sucrose in the amount of 5% or more may often be indicative of the early harvest of honey from a hive [42].

### 2.3. The Floral and Geographical Variations in Honey Based on Sugars

This study focuses on summarizing the variation in the sugar composition of honey produced worldwide. In 2018, Pascual-Mate et al. [46] conducted a study focused on the ripeness-related and crystallization aspects of honeys from the Northern Iberian Plateau in central Spain. The research involved the analysis of 54 artisanal honey samples obtained directly from beekeepers’ hives in 2011. These honeys were made from multifloral sources with an average of 18 different pollens identified per sample. The study identified between 37.53 and 39.93% fructose, 26.86 and 31.03% glucose, and 0.1 and 1.54% sucrose. The disaccharides and trisaccharide identified in this study were in small quantities, between 0.1 and 5% in all the samples. Overall, the total percentage of sugar reported in this study ranged between 71.95% and 77.68%, while the sum of the percentages of fructose and glucose was between 63.1% and 70.96%. The experiments were carried out using gas chromatography (GC). In another study carried out in Turkey to investigate the physical and chemical properties of honey, the sugar composition of honey from 13 different unifloral sources and 1 multifloral source was analyzed and compared per 100 g of honey sample [47]. The unifloral sources included heather, rhododendron, chestnut, lavender, Jerusalem tea, chaste tree, astragalus, clover, common eryngo, and acacia. The percentage of fructose found in these samples ranged from 28.30 to 45.11%, while glucose was found to be within the range of 17.40–25.9%. The highest amount of fructose was found in heather floral sources, and the least was observed in acacia. Honey from chaste tree sources contained the highest concentration of glucose, whereas that from clover plants showed the lowest amount. The amount of sucrose present in these samples was below the detection limits of 3.39%. Out of the 13 unifloral honeys analyzed in the study, no sucrose content was detected in 10 of the samples. Sucrose concentrations were detected in the honey from chestnut, astragalus, and acacia. One other sample studied in this analysis was from a multifloral source and showed a sucrose concentration of approximately 1%. Apart from sucrose, three other disaccharides, maltose, trehalose, and melibiose, were also analyzed in this study. The concentrations of these sugars in the honey samples were all below 0.7%. The trisaccharide melezitose was also detected in all the samples. The percentage of the concentration of melezitose was within the range of 0.5% to 1.0%. The sum of fructose and glucose for the 14 samples ranged between 51% and 71%. The fructose/glucose ratios calculated in this work were between 1.19 and 1.98. The researchers reported that ribose, galactose, and arabinose were not present in any of the samples analyzed in this study. The analyses of the sugars were performed using high-performance liquid chromatography with a refractive detector (HPLC RID).

We also considered honey from Brazil and Venezuela in South America. In Brazil, unifloral honeys produced by the stingless bees Jadaira (M. Subnida Duke) and uruçu (*Melipona scutellaris* Latrelle) in specific semi-arid regions of the northeastern part of Brazil were analyzed. These regions included Juazeiro, Malícia, Velame, Branco, and Jurema Branca. The amounts of glucose, fructose, and sucrose measured in the honeys produced by the Jadaira bees ranged from 37.7 to 45.7 g/100 g, 49 to 56 g/100 g, and 0.7 to 4.1 g/100 g, respectively [48]. The honeys produced by the uruçu bees also showed ranges of 27.8–43.4 g/100 g, 52.9 g/100 g–59.4 g/100 g, and 1.0 g/100 g–3.5 g/100 g for glucose, fructose, and sucrose, respectively. Maltose was not detected in any of the samples. The amount of arabinose measured in all the samples ranged from below the detection limit to 1.1 g/100 g sample. The total sugars measured in the samples produced by the Jadaira bees were from 62.4 g/100 g to 72.6 g/100 g, and those by the uruçu were 62.3 g/100 g–72.7 g/100 g. The F/G ratios observed in this study were between 1.1 and 1.7 and 1.2 and 1.5 for the Jadaira honey and urucu honey, respectively. Similar to the earlier studies described, these experiments were also carried out by HPLC equipped with a refractive detector.

The samples from Venezuela were analyzed in 1996 to establish their sugar profile as part of the quality criteria for distinguishing between honey types. The study analyzed 42 stingless bee honeys from different entomological sources. Of these samples, 24 were honey samples from three different melipona species (melipona compressipes, melipona trinitatis, and melipona favosa) of honeybees, 8 were samples from frieseomelitta aff varia, and the remaining 10 were from other non-melipona species. The honeys from the melipona species of honeybees did not show significant differences in their concentrations of fructose. The fructose concentrations for this group of honeys ranged from 32.3 to 38.6 g/100 g, whereas the frieseomelitta and other non-melipona species were 20.3–28.5 g/100 g and 20.4–28.4 g/100 g, respectively. The glucose samples were within the range of 33.5–39.6 g/100 g for the melipona honeys, 11.2–20.0 g/100 g for the frieseomalitta honeys, and 17.8–35.0 g/100 g for the other non-melipona honeys. The concentration of sucrose in all the samples analyzed was between 0.1 and 0.2 g/100 g. The other disaccharides measured in the study, such as turanose, maltose, and trehalose, ranged from below detection limits to 32.3 g/100 g. Erlose was the only trisaccharide measured in the study. The concentrations determined ranged from below the limit of detection to 0.5 g/100 g [49].

Across Africa, analyses of the sugar profile of honeys from Algeria and Ethiopia have also been carried out. Between 2004 and 2006, Ouchemoukh and his group collected fifty samples from different regions in Algeria. The study found a total of 10 sugars in the samples examined, comprising 2 monosaccharides, 5 disaccharides, and 3 trisaccharides. The quantities of sugars, ranked from highest to lowest, were fructose, glucose, maltose, saccharose, turanose, isomaltose, erlose, melezitose, raffinose, and trehalose. The percentage of fructose was in the range 35.99–42.57%, and glucose was in the range 24.63–35.06%. Overall, the total sugars content of the samples analyzed was between 73.05 and 81.38%. The honeys from multifloral sources showed consistent total sugar levels of about 76%. The F/G ratio was found to be between 1.11 and 1.36 [50]. In an analyses of Ethiopian honeys, 320 samples of honeys from monofloral sources were investigated. The study identified six different sugars, which included fructose, glucose, sucrose, maltose, turanose, and isomaltose. The highest concentration of fructose per 100 g was determined to be in the honey from Acacia floral sources (43.1 ± 0.4 g), and the lowest was 35 ± 4 g/100 g from Becium grandiflorum. The researchers also found the glucose concentration range to be between 37.2 ± 0.4 g/100 g and 29 ± 3 g/100 g in Leucas abyssinica honey. The amount of sucrose found in this study ranged between 1.11 and 3.40 g/100 g. The sugar content of the honey ranged from 72.4 to 79.7 g/100 g, primarily consisting of monosaccharides [51].

In this review, we also summarize the sugar profile of honeys produced in some parts of Asia. In the first of these studies, 58 honey samples from Oman, obtained from 18 different geographical area, were analyzed. These samples were harvested in 2016. The winter floral honey samples analyzed had an average of 22.7% glucose, whereas the summer floral samplessamples had an average of 25.3% and 28%, respectively. The fructose concentration determined in this study was 34.9% for the summer samples, 31.6% among the winter samples, and 35.6% for the multifloral samples. Also, the percentage of sucrose determined in the study was 0.1–17.5%, 0–2.77%, and 0–2.84% for the winter, summer, and multifloral samples, respectively. The total percentage of sugar present in this study averaged between 42.1 and 71.3%. This analysis was carried out using ion chromatography with an electrochemical detector [52]. In another study, Kek and their research group also analyzed the fructose, glucose, and sucrose contents of honeys from Malaysia in 2017. Five different types of Malaysian honey samples, tualang, gelam, pineapple, Borneo, and Kelulut, were investigated in this study. The analyzed samples were collected from January 2013 to March 2014. The fructose content was in the range of 39.92–48.44 g/ 100 g, glucose was within the range of 26.72–41.96 g/100, and sucrose was less than 1 g/100 g. The sum of fructose and glucose in the honeys was found to be 75.16–81.93 g/100 g [53]. In Saudi Arabia, another study was carried out using GC-MS to determine the total sugar content present in 14 honey samples collected from the Saudi Arabian market. The study reported a fructose concentration range between 29.08 and 39.48%, a glucose concentration range between 2.02 and 34.10%, and the amount of sucrose determined was below the detection limit of 2.95%. The total sugar content determined by this study was found to be between 50.26% and 74.74% [54].

Also, in Australia, the amount of fructose and glucose in five different honey samples were determined and their respective F/G ratios were calculated. The amount of fructose determined to be in these commercial samples ranged from 387 mg/g to 439 mg/g or 38.7 g/100 g to 43.9 g/100 g. The glucose concentrations were also between 249 mg/g and 337 mg/g or 24.9 g/100 g and 33.7 g/100 g. In this research, the F/G ratios were between 1.3 and 1.6. This study was carried out using high-pressure thin-layer chromatography [55]. Table 2 summarizes the various studies that have been reviewed.

According to the *Codex Alimentarius*, the combined amount of fructose and glucose in honey samples should be at least 60 g/100 g, except for honeydew honey and mixtures of honeydew honey with blossom honey, which should not be less than 45 g/100 g [56]. Also, honey’s sucrose concentration should generally not exceed 5 g/100 g, with some exceptions. Honey from specific plants, such as alfalfa, citrus, false Acacia, French honeysuckle, Menzies Banksia, red gum, leatherwood, and Eucryphia milligani, should not exceed 10 g/100 g. Additionally, honey from lavender and Borage must have a sucrose concentration of less than 15 g/100 g. It does appear that most of the sugar analyses covered in this review were within the limits set by the *Codex Alimentarius*. While some of the researchers acknowledged that some of the samples deviated from the *Codex* standards, some authors argued for the genuineness of the honey, and proposed that the findings could be used as a basis for updating the existing standards [47].

This paper focuses on identifying the polyphenolic and sugar contents of the honeys that may be found in the U.S. honey market to establish their authenticity. However, from our literature searches, it appears that extensive work on a global scale to establish the reliable sugar contents of honey from most geographical areas has not been carried out. The fact that most adulterants in honey are sweeteners necessitates further inquiries into honey sugars to give consumers and honey industry players in the U.S. extensive data for the easy identification of adulterated honey that may be imported into the country.

**Table 2:** Summary of analyses of sugars in honeys across five continents.

Continent	Country	Percentage of Identified Sugars	Total Sugar	Methods	Continent	Country	Percentage of Identified Sugars	Total Sugar
		Fructose	Glucose	Sucrose	Trs	Diss		
Europe	Spain	37.53–39.9	26.86–31.03	0.1–1.54	0.1–1.54		71.95–77.68	GC [57]
	Turkey	28.30–45.11	17.40–25.90	b/d–3.39	0.5–1.0	b/d–0.7	51–72	HPLC-RID [58]
South America	Brazil	27.8–45.70	49.0–59.4	0.7–4.10	N/A	N/A	62.4–72.7	HPLC [59]
	Venezuela	20.30–38.60	11.20–39.60	0.1–0.20	b/d–0.50	b/d–32.3	74.7–76.3	HPLC [60]
Africa	Algeria	35.99–42.57	24.63–35.06	0–5.26	0.00–1.14	0.01–1.63	73.05–81.38	HPAEC-PAD [61]
	Ethiopia	31.77–43.40	26.0–37.55	1.11–3.40	N/A	N/A	72.4–79.7	HPLC-RI [62]
Asia	Oman	31.60–35.60	22.70–28.00	0.11–0.15	N/A	N/A	42.1–71.3	IC [63]
	Malaysia	39.92–48.44	26.72–41.96	<1.00	N/A	N/A	75.16–81.93	HPLC-ELSD [64]
	Saudi Arabia	29.08–39.48	20.02–34.10	b/d–2.95	N/A	N/A	50.26–71.3	GC-MS [65]
Australia	Australia	38.7–43.9	24.9–33.7	N/A	N/A	N/A		HPTLC [66]

b/d refers to below detection; N/A means those sugars that were not studied in the research; Trs and Diss are trisaccharides and disaccharides, respectively.

### 2.4. Geographical Markers Identified in Honey Based on Polyphenols

The origin of honey is essential for predicting its chemical composition, organoleptic characteristics, health, and nutritional quality. Although the chemical makeup of honey is primarily linked to its botanical origin, it also depends to some extent on the location where it was produced. This is because the environment, including the soil and climate conditions, plays a role in shaping the types of flowering plants that bees forage for nectar [67]. The determination of honeys’ geographical origins has become a vibrant field, employing chemometric classification procedures. This has been driven by the realization that relying solely on the botanical source of a honey may not provide sufficient information about its identity. Several studies aligned with key global honey-producing regions have addressed the concept of geographical origin in their studies [24,68,69,70,71]. Ensuring the authenticity of honeys’ geographical origins is a crucial aspect of quality control and food safety, given the significant variation in international honey prices that are tied to its source. Recently, there has been increased interest aimed at authenticating the origins of various honeys through the identification of specific compound markers based on polyphenols.

In this review, we have investigated the polyphenol markers in honeys originating from diverse geographical sources, focusing on the popular honey-producing regions in Europe, Asia, and Africa. The comprehensive study conducted by Sergiel et al. [30] introduced an HPLC-MS/MS method to characterize Polish honeys based on their phenolic compound content. The analysis revealed various phenolic acids, including ferulic, caffeic, p-hydroxybenzoic, chlorogenic, p-coumaric, and vanillic acids, along with flavonoids, like kaempferol, luteolin, rhamnetin, rutin, and quercetin, in the honeys. Although tricetin and genistein were not found, hesperetin was detected in heather and linden honeys. Additionally, rutin was identified in rape honey for the first time. In a study focused on Polish monofloral goldenrod honey, gallic acid, 4-hydroxybenzoic acid, and p-coumaric acid were identified as the main phenolic components, both in the honey and the flowers [72]. Honeydew honey, as revealed in various studies, exhibits substantial concentrations of protocatechuic acid. This specific compound has been identified as a distinguishing factor in conifer tree honey (from pine and fir) from Greece [73]. Greek honey samples, including thyme honey, displayed the presence of phenolic acids, such as p-hydroxybenzoic acid, vanillic acid, caffeic acid, and p-coumaric acid, with p-hydroxybenzoic acid identified as the dominant compound [73]. Similarly, protocatechuic acid was also found in significant amounts in pine and oak honeydews from Turkey. Ellagic acid, on the other hand, was detected in substantial amounts exclusively in oak honeydew produced in Turkey [74]. Additionally, p-coumaric acid, ferulic acid, and hydroxycinnamates caffeic were detected as floral markers in chestnut, sunflower, lavender, and acacia honeys from Italy, France, and Germany, and in unifloral honeys from other European regions [75]. Ellagic acid was identified simultaneously in this study as a potential floral marker for heather honeys from England and the Netherlands. A phenolic profiles analysis of Spanish honeys demonstrated that the botanical origin of honey significantly influences the composition of flavonoids and phenolic compounds, allowing for differentiation based on the prevalence of specific compounds. For instance, citrus honey is characterized by hesperetin; rosemary honey by chrysin, pinocembrin, kaempferol, caffeic acid, and naringenin; and honeydew honey by myricetin, quercetin, galangin, and p-coumaric acid [66]. Trans- and cis-abscisic acids are known to be unique geographical markers for Portuguese heather honey [67].

In Asian honeys, specifically Chinese buckwheat honey, rutin, p-coumaric acid, and p-hydroxybenzoic acid were identified, with slightly higher contents compared to honey from Poland [30] and Italy [68]. The chemometric tools used for the classification showed that Chinese rapeseed honey had elevated levels of benzoic acid, pinocembrin, kaempferol, and naringenin [70]. An analysis of distinct unifloral honey samples from India, including eucalyptus, lemon, neem, and ginger, indicated the presence of several phenolic compounds, namely gallic acid, caffeic acid, *p*-coumaric acid, chlorogenic acid, ellagic acid, and ferulic acid [76]. The concentrations of caffeic acid and chlorogenic acid were consistent with those of previous findings for Malaysian honeys [65]. Similarly, the levels of *p*-coumaric acid and chlorogenic acid were comparable to those reported in citrus, rosemary, chestnut, and sunflower honeys from various European regions [77]. In a previous study, the phenolic acid and flavonoid composition of eight Malaysian honey types, such as acacia, pineapple, gelam, longan, Borneo, rubber tree, sourwood, and tualang honeys, were thoroughly analyzed. The study focused on identifying and quantifying thirteen specific phenolic compounds, which included seven flavonoids and six phenolic acids. The study showed that longan and tualang honeys exhibited a higher quantity of phenolic compounds compared to acacia, Borneo, and rubber tree honeys, which had a lower concentration of these compounds. Benzoic acid was shown to be the most abundant phenolic acid in the analyzed Malaysian honeys, followed by caffeic acid, catechin, myricetin, gallic acid, and naringenin [73]. In an analysis of monofloral honey samples collected from various districts in Bangladesh, the most prevalent flavonoids identified were kaempferol, catechin, myricetin, and naringenin, while the dominant phenolic acids included gallic acid, caffeic acid, chlorogenic acid, benzoic acid, and trans-cinnamic acid. In this study, the researchers also provided essential data on the proline, phenolic acids, and flavonoids contents, contributing to the substantiation of medicinal claims associated with different honey types in the country [74]. The mean values for the total phenolic acids, total flavonoid content, and proline content were 199.20 ± 135.23 mg/kg, 46.73 ± 34.16 mg/kg, and 556.40 ± 376.86 mg/kg, respectively. In a separate study focused on identifying and characterizing phenolic compounds in honey from Megamalai, Theni District, Tamilnadu, in India, five distinct phenolic acids were detected, with gallic acid and rutin being the most abundant. This study highlighted the increased levels of phenolic acids and flavonoids in the Megamalai honey sample, suggesting its potential as a rich source of antioxidants and antibacterial properties [75].

This review has extensively examined polyphenol markers in African honeys, focusing on samples from various geographic regions. In an analysis of genuine samples of cotton, clover, and citrus honeys from various regions in Egypt, it was found that citrus and clover honey samples contained a total of 18 phenolic compounds. However, cotton honey samples showed a slightly higher number, with 20 phenolic compounds detected. Among the different types of honey, citrus and clover honeys were dominated by caffeic acid, Ber el abed honey had galangin as the predominant compound, and Siwa honey contained gallic acid in significant amounts [78]. Ouchemoukh et al. [79] characterized the phenolic profiles of Algerian honeys, analyzing 35 honey samples from different regions of Algeria. The study identified more than 30 compounds in the honey samples, including 14 phenolic acids and 16 flavonoids, expanding the knowledge of the phenolic composition of Algerian honeys. Specific compounds, like 4-hydroxybenzoic acid, apigenin, kaempferol, isorhamnetin, luteolin, pinocembrin, etc., were found in all the honey extracts. The study highlighted potential floral markers, such as caffeic and p-coumaric acids for Capparis spinosa and Trifolium honeys, respectively. Additionally, an analysis of Tunisian honeys uncovered a new flavonoid, myricetin 3,7,4′,5′-tetramethyl ether, and a minor compound, quercetin 3,7,3′-trimethyl ether, present in propolis in high amounts and in honey in smaller quantities [60].

An examination of the phenolic compounds and methylglyoxal in manuka and kanuka honeys from New Zealand revealed that both types share six primary phenolic acids, albeit with varying relative proportions. Manuka honey exhibited higher concentrations of trimethoxybenzoic acid and methylglyoxal, and there was a linear correlation between 2-methoxybenzoic acid and methylglyoxal in fresh manuka honey. In contrast, kanuka honey exhibited a higher level of methoxyphenyllactic acid. The study found that the phenolic components in both types of honey increased as they matured [80]. Goslinski et al. [81] conducted a comparative study that revealed significant variations in the polyphenolic profiles of domestic honeys from Poland and imported honeys from New Zealand and Malaysia. Manuka and Malaysian honeys were characterized by high concentrations of caffeic acid, datiscetin, and chlorogenic acid, ranging from 35.69 to 38.67 mg/kg, 2.09 to 2.75 mg/kg, and 2.68 to 3.29 mg/kg, respectively. Caffeic acid emerged as the most abundant phenolic compound, followed by p-coumaric acid, chlorogenic acid, quercetin, and datiscetin, which were present at lower levels in all the samples. A comprehensive list of polyphenol-based authenticity markers identified in honey from diverse global geographical and botanical origins is presented in Table 3.

The major polyphenols reported in honey are flavonoids. Numerous studies have established a connection between the flavonoid profiles of honey and their geographical origins. Pinocembrin, chrysin, pinobanksin, and galangin were reported as major flavonoids in Portuguese heather honey [76]. The flavonoid profile of Portuguese honeys indicated that myricetin, myricetin 3-methyl ether, myricetin 3′-methyl ether, and tricetin could be used as distinct markers to determine their floral origin. Luteolin was suggested as floral marker for lavender honey [65]. An analysis of Spanish honeys showed a flavonoid composition comparable to that found in honey from different locations. The previously identified main flavonoid compounds in Spanish honey include pinocembrin, pinobanksin, galangin, chrysin, luteolin, apigenin, isorhamnetin, and quercetin 3-methyl ether [77]. A previous work by Tomas-Barberan indicated the presence of flavonoids, such as quercetin, hesperetin, kaempferol, and 8-methoxykaempferol, as candidate floral markers for honey samples from different European countries, including Germany, France, Denmark, Spain, Italy, and Portugal [65]. Eucalyptus honey from Tunisia was characterized by ellagic acid [82]. On the other hand, rosemary and orange Tunisian honeys contained 8-kaempferol and hesperetin, respectively. The predominant flavonoids identified in different honey samples from India were naringenin, kaempferol, pinocembrin, chrysin, and quercetin [83]. In a previous study, the impact of floral and geographical factors on the phenolic composition of honey from different countries, such as China, New Zealand, Spain, Brazil, Canada, Germany, and Italy, were investigated. Quercetin, apigenin, hesperitin, kaempferol, chrysin, and luteolin were identified as the most abundant flavonoids, with concentrations varying widely among the honey samples. The result of this study highlighted the usefulness of flavonoid markers for the authentication of a specific honey’s origin [19].

**Table 3:** Polyphenol markers identified in honeys from popular geographical destinations around the globe.

Geographical Location by Continent	Honey Type	Proposed Markers	Analytical Detection Method	Reference
Europe				
Macedonia	Acacia	Naringenin, caffeic acid	HPLC-DAD-MS	[84]
Macedonia and Bulgaria	Chestnut	Kynurenic acid		
	Multifloral	Naringenin, caffeic acid		
Italy, France	Chestnut and floral honey	Quercetin	HPLC-DAD	[85]
Italy	Strawberry tree honey	Homogentisic acid	HPLC-MS	[86]
			RP-HPLC-UV	[87]
			HPLC-MS/MS and Q-TOF	[88]
Greece	Multifloral	Quinic acid, gentisic acid, 3-(2,5-dimethoxyphenyl) propanoic acid	UPLC-QTof-MS	[89]
Poland		Chrysin, acacetin, sebacic acid, isorhamnetin		
Spain	Lavender	Luteolin, naringenin	Micellar electrokinetic capillary chromatography	[90]
	Citrus	Hesperetin	Capillary zone electrophoresis	[91]
	Thyme	Rosmarinic acid		
	Heather	Ellagic acid		
Ukraine, Hungary	Buckwheat	3-hydroxybenzoic acid, ferulic acid	LC-DAD	[92]
Croatia	Mint	Methyl syringate		
	Sage (*Salvia officinalis* L.)	Lumichrome		
	Savory	Methyl syringate		
Italy, Croatia, France	Sweet chestnut	Kynurenic acid	LC-DAD	
Portugal	Heather	Myricetin, myricetin-3′-methyl-ether, myricetin-3-methyl ether, tricetin	HPLC-DAD	[93]
Spain	Rosemary	8-methoxykaempferol	HPLC-DAD	[94]
		Kaempferol		
England, France, Germany, Denmark	Rapeseed	Cis–trans-abscisic acid, trans–trans-abscisic acid, quercetin, 8-methoxykaempferol, kaempferol	HPLC-DAD	[67,76,95]
Italy, Portugal, Spain	Eucalyptus (eucalyptus carmaldulensis)	quercetin		
Germany, France, the Netherlands, England	Heather	Ellagic acid, cis–trans-abscisic acid, trans–trans-abscisic acid		
Spain, Portugal	Rosemary	8-methoxykaempferol, kaempferol		
Turkey	Chestnut	p-hydroxybenzoic acid, caffeic acid		
Germany, Italy, France	Acacia	Cis–trans-abscisic acid, trans–trans-abscisic acid		
Italy, Slovakia	Acacia	Kaempferol rhamnosides	HPLC-DAD-MS/MS	[96]
Serbia	Sunflower	Quercetin, eriodictyol	UHPLC-MS (LTQ and Orbitrap)	[97]
	Acacia and linden honey	Cis-, trans-abscisic acid		
Italy	Thistle honey	Lumichrome, phenyllactic acid	HPLC-DAD-MS/MS and Q-TOF	[98]
	Asphodel honey	Methyl syringate	HPLC-DAD, LC-MS/MS	
Multiple European regions	Chestnut	4-hydroxybenzoic acid, Dl-p-hydroxyphenyllactic acid, ferulic acid, phenylacetic acid	HPLC-UV	[72]
	Heather	Benzoic acid, phenylacetic acid, L-β-phenyllactic acid		
	Lime honey	3-hydroxybenzoic acid		
	Eucalyptus	Benzoic acid derivatives		
Serbia	Buckwheat, oilseed rape, and goldenrod honey	Chlorogenic acid, dicaffeoylquinic acid	UHPLC-MS	[97]
Finland	Lingonberry and mire honey	Cinnamic acid, p-OH-cinnamic acid, acacetin	HPLC-MS/DAD	[99]
Spain	Heather	4-anisaldehyde, 3-hydroxyphenylacetic acid, 4-hydroxybenzaldehyde	LC-MS/MS	[32]
**Asia**				
China	Buckwheat honey	Rutin, p-coumaric acid, p-hydroxybenzoic acid	HPLC-MS/MS	[69]
	Rapeseed honey	Benzoic acid, kaempferol, naringenin, pinocembrin		
	Sunflower honey	Quercetin, chrysin		
	Codonopsis honey	Gallic acid		
China	Citrus	Caffeic acid, p-coumaric acid, ferulic acid, hesperetin	HPLC-ECD	[100]
China	Longan	Syringic acid, p-coumaric acid, ferulic acid	HPLC-ECD	[101]
China	Vitex honey	Chlorogenic acid, 4-hydroxybenzoic acid, luteolin, caffeic acid, 3-hydroxybenzoic acid	UHPLC-MS/MS	[102]
China	Rapeseed (*Brassica rapa Linn*)	Ellagic acid	LC-ECD	[103]
	Linden honey	3,4-dihydroxybenzoic acid, isorhamnetin, gallic acid	UHPLC-MS/MS	
China	Astragalus honey	Calycosin, isorhamnetin	UHPLC-MS/MS	[102]
	Codonopsis honey	Myricetin, rutin, gallic acid, formononetin	UHPLC-MS/MS	
China	Wild Chrysanthemum, jujube flower, acacia	Gallic acid, chlorogenic acid, caffeic acid, syringic acid, protocatechuic acid, rutin	HPLC-DAD	[104]
China	Rape and chaste honey	Kaempferol, morin, ferulic acid	HPLC-DAD-MS/MS	[105]
Malaysia	Apis honey	chrysin	HPLC-DAD	[106]
	Gelam honey	Gallic acid, ellagic acid		
	Starfruit honey	Salicyclic acid, benzoic acid, 4-hydroxybenzoic acid		
Malaysia	Longan and tualang	Catechin, caffeic acid, myricetin, gallic acid, naringenin	HPLC-PDA	[107,108]
	Acacia honey	Ferulic acid		
Bangladesh	Mustard honey	Caffeic acid, benzoic acid, kaempferol, gallic acid, myricetin, apigenin	HPLC-UV	[74]
India	N/A	Catechin, ferulic acid, rutin	HPLC-UV	[109]
Yemen	Ziziphus Spina-Christi honey	Gallic acid, 4-hydroxybenzoic acid, 4-hydroxyphenylacetic acid, vanillic acid, galangin	HPLC-DAD, UHPLC-MS	[110]
**South and North America**				
Ecuador	Stingless bee honey	Luteolin	HPTLC	[111]
Brazil	Jandaira honey	Naringenin, isorhamnetin, vanillic acid, gallic acid, cinnamic acids	HPLC-DAD	[112]
Brazil	Japanese grape honey	Gallic acid, p-coumaric acid	HPLC-UV	[113]
	Eucalyptus	Gallic acid		
	Mastic honey			
	Polyfloral			
Cuba	Linen vine honey	p-coumaric acid, ferulic acid, quercetin	HPLC-DAD-MS/MS	[114]
	Morning glory honey	Ferulic acid, p-coumaric acid		
	Black mangrove	p-coumaric acid, kaempferol		
	*M. Beecheii honey*	C-pentosyl-C-hexosyl-apigenin	HPLC-DAD	[115]
Cuba	*Apis Mellifera*	Caffeic acid, ferulic acid		
USA	Multifloral honey	Apigenin, pinocembrin, myricetin, 6-phenylnarigenin, kaempferol, ferulic acid-5,5-dihydroferulic acid	HPLC-MS/MS	[31]
USA	Peppermint honey	p-coumaric acid, kaempferol	HPLC-DAD	[115]
**Middle East**				
Morocco and Palestine	Multifloral	Gallic acid, tannic acid, pyrogallol, coumaric acid	HPLC	[116]
Iran	Citrus honey	Quercetin, hesperetin, chrysin	MLC-UV	[117]
Pakistan	*Sidr honey* (*Ziziphus species*)	Caffeic acid, chlorogenic acid, ferulic acid	Florescence spectroscopy	[118]
**Africa**				
Mozambique	Multifloral	Pinocembrin, kaempferol, rutin, catechin	HPLC-PDA	[119]
Algeria	Capparis Spinosa, Trifoliumn honey	Caffeic acid, p-coumaric acid	LC-DAD	[79]
Sudan	Sunflower, blue nile, sunnut, Tahil honey	Hesperetin, apigenin, kaempferol, isorhamnetin	HPLC-PDA	[120]
Egypt	Cotton honey	Hesperetin, quercetin, cinnamic acid, p-hydroxybenzoic acid	HPLC-UV	[121]
Egypt	Clover and citrus	Caffeic acid	HPLC-UV	[78]
Tunisia	Eucalyptus	Ellagic acid, quercetin, kaempferol	HPLC-DAD	[60,82]
	Rosemary	Kaempferol, 8-methoxykaempferol		
	Thyme	Kaempferol, isorhamnetin		
	Rape	Chrysin		
**New Zealand and Australia**				
New Zealand	Manuka honey	Trimethoxybenzoic acid, methylglyoxal, 2-methoxybenzoic acid	HPLC-MS	[80]
	Kanuka honey	4-methoxyphenyllactic acid		
New Zealand	Leptospermum manuka	Quercetin, luteolin, quercetin-3-methyl ether	HPLC-DAD	[122]
	Blackberry	Kaempferol, quercetin, chysin, trans-ferulic acid		
Australia	Crow ash (*Guioa semiglauca*) honey	Myricetin, tricetin, quercetin, luteolin	HPLC-PDA	[59]
Australia	*Australian sunflower honey, tea tree, heath honey*	Quercetin, myricetin, luteolin	HPLC-PDA	
Australia	Eucalyptus camaldulensis (river red gum)	Tricetin	HPLC-DAD	[123]
	Eucalyptus pilligaensis (mallee honey)	Luteolin		

### 2.5. Characterization of Polyphenol Markers in U.S. Honey

Since polyphenolic compounds play a pivotal role in honey authentication, we explored the few cases that have leveraged polyphenol profiles to successfully identify and authenticate U.S. honeys. Marshall and coauthors examined the polyphenol profiles of twenty honey samples from various geographical regions in Florida to identify U.S. honeys. The findings showed the presence of nine phenolic compounds, including coumaric acid, kaempferol, quercetin, rutin, luteolin, pinocembrin, and the plant hormone 2-trans,4-trans-abscisic acid, in the Florida honeys [124]. Interestingly, the quantitative levels of the individual phenolic compounds were reported to be comparatively lower (<1 μg/g) than those documented in other studies. While specific markers for the honeys were not identified, the study suggested that the floral and production origins of the honeys significantly contributed to their health-promoting properties. The phenolic compounds and their content identified in the Florida honey varieties are summarized in Table 4.

**Table 4:** Phenolic compounds detected in U.S. honeys using different analytical techniques.

Geographical Origin	Floral Origin	Total Phenolic Content (µg/g)	Phenolic Compounds Identified	Analytical Technique	Reference
USA	Buckwheat	46.5 ± 0.57	Vanillic acid, p-coumaric acid Syringic acid, p-hydroxybenzoic acid Cinnamic acid, cis-, trans-abscisic acid Pinobanksin, pinocembrin Galangin, chrysin Kaempferol, quercetin	HPLC-DAD-MS	[125]
	Clover	33.1 ± 2.23			
	Acacia	11.6 ± 0.95			
	Fireweed	14.8 ± 2.55			
	Tupelo	20.9 ± 1.93			
	Soy	50.7 ± 3.0			
	Hawaiian Christmas berry	1.96 ± 0.29			
USA	Gallberry	33.1–55.0	Quercetin; luteolin; pinocembrin; chrysin; galangin; rutin; coumaric acid; 2-trans,4-trans-abscisic acid; 2-cis,4-trans-abscisic acid	HPLC-DAD-MS	[124]
	Citrus	12.2–47.6			
	Palmetto	19.5–43.7			
	Tupelo	15.1–55.8			
	Manuka	7.34–13.5			
	Multifloral	6.27–102			
USA	Clover		p-hydroxybenzoic acid; vanillic acid; syringic acid; p-coumaric acid; cis-, trans-abscisic acid cinnamic acid; pinobanksin; quercetin; pinocembrin; kaempferol; chrysin; galangin	HPLC-DAD	[126]
USA	Buckwheat		p-hydroxybenzoic acid; vanillic acid; p-coumaric acid; cis-, trans-abscisic acid; cinnamic acid; pinobanksin; pinocembrin; kaempferol; chrysin; galangin	HPLC-DAD	[126]
USA	Multifloral	N/A	Isorhamnetin, quercetin, kaempferol, naringenin, chysin, apigenin, pinocembrin, hesperidin, ferulic acid, caffeic acid, 5′5-dihydroferulic acid, sinapic acid, 6-phenylnaringenin	HPLC-MS/MS	[31]

In response to the limited data on the phenolic profiles of honey in North America, particularly the United States, Gheldof et al. [125] conducted a study to explore the phenolic composition of seven distinct monofloral honey types and assess their potential antioxidant activity. A variety of phenolic compounds were present in the majority of the honey samples, including *p*-coumaric acid, cis- and trans-abscisic acid, *p*-hydroxybenzoic acid, cinnamic acid, pinobanksin, pinocembrin, and chrysin. Table 4 provides a summary of the phenolic acids and flavonoids described in this study. The results from this study provided insights into the diverse phenolic profiles of U.S. honeys and their antioxidant potential.

In a recent study, the polyphenolic profiling of honey samples from multiple geographical locations in the United States detected the presence of eighteen distinct phenolic compounds. These compounds included flavonoids, such as quercetin-3-O-(6-malonyl-glucoside), naringenin, chrysin, apigenin, pinocembrin, 6-phrenylnarigenin, hesperidin, kaempferol 3-O-rhamnoside, isorhamnetin, quercetin, kaempferol, and (+)-catechin 3-O-glucose, along with five phenolic acids—sinapic acid, caffeic acid, 5,5-dihydroferulic acid, ferulic acid, and subaphyllin. This study was the first to report the presence of these compounds in the selected sample locations, adding novel insights into the polyphenolic composition of U.S. honey. A polyphenol screening analysis further unveiled twelve polyphenolic markers, with kaempferol, caffeic acid, myricetin, kaempferol-3-O-rhmnoside, and (+)-catechin-3-O-glucose identified as the dominant markers in honeys originating from Washington, Texas, Idaho, Utah, and Colorado, respectively [31].

Wang et al. [127] examined the influence of heat and storage conditions on the antioxidant capacities and polyphenolic contents of clover and buckwheat honey originating from the United States. While processing had no significant effect on the clover honey, it led to a 33.4% reduction in the antioxidant capacity of the buckwheat honey. Twelve phenolic compounds were identified in the clover and buckwheat honey, although a few exceptions were found. Syringic acid and quercetin were absent in the buckwheat honey. The phenolic compounds identified in the raw and processed U.S. domestic clover and buckwheat honeys can be found in Table 4. Altogether, our research on the U.S. honey market shows that reliable authenticity markers for U.S. honeys remains underexplored, highlighting the need for region-specific research on U.S. honeys.

### 2.6. Analytical Approaches to Polyphenol Profiling in Honey

To enhance the authentication of honey as coming from various botanical and geographical origins, modern analytical methods have been employed over traditional detection techniques. The classic methods for honey authentication are characterized by several drawbacks, including being time consuming, tedious, and less reliable for an accurate determination [128]. Cutting-edge analytical tools, like chromatography and mass spectrometry, infrared and Raman techniques [33], nuclear magnetic resonance spectroscopy, stable isotope analysis [129], and flame ionization detectors [130], have been employed for assessments of honey quality. These methods provide a more reliable and comprehensive understanding of honey’s characteristics, ensuring a more accurate and rapid determination of its origin. Spectrophotometric techniques, known for their fast and non-invasive nature, are extensively employed for assessing honeys’ phenolic constituents [43]. This characteristic positions them as an environmentally friendly analytical alternative. However, the utilization of chemometric tools has proven effective for the reliable processing and classification of honey origins [131]. Chromatographic techniques, on the other hand, serve a crucial role in assessing the authenticity of honey by establishing a chromatographic profile, often referred to as a fingerprint. High-performance liquid chromatography (HPLC) is commonly used for characterizing the phenolic compounds when coupled with mass spectrometry (MS) detection. The process of analyzing the phenolic compounds of honey comprises various stages: the extraction process, analytical separation, identification, and quantification [132]. Common extraction procedures typically include solid-phase extraction (SPE) or liquid–liquid extraction (LLE), while the adoption of ultra-high-performance liquid chromatography has led to improved resolution, sensitivity, and faster analysis. Various detection systems are employed in HPLC instrumentation, including UV detectors, diode-array detectors (DADs), electrochemical detection systems, and mass detectors, including multi-stage MS. These detection systems have proven successful at achieving improved resolution and sensitivity [133]. In recent studies, the utilization of chemometric methods and fingerprinting have allowed for the detection of phenolic markers for the authentication of honey from various origins. For instance, polyphenolic fingerprinting and chemometric methods were utilized to unveil twelve geographic markers of honey from different regions in the United States [31]. By employing multivariate chemometric techniques, Wen and his group found that rapeseed honey from China was distinct from other Chinese unifloral honeys (sunflower, buckwheat, and *Codonopsis*) [69]. A principal component analysis (PCA) played a key role in highlighting these differences. Furthermore, the identification of potential floral markers, such as benzoic acid and kaempferol, using classification tools contributed to the differentiation among honey varieties. The researchers successfully identified 18 phenolic compounds and achieved a 94% correct prediction rate using a linear discriminant analysis (LDA) model. In a separate investigation, Giordano et al. [131] utilized FTIR spectra to distinguish the floral origins of monofloral honey samples from Chile. The study involved forty-nine honey samples sourced from various geographical regions, and their botanical origins were determined through a melissopalynology analysis. By employing a multivariate discriminant analysis approach, the study further showed that Quillay, Corcolén, and Tebo honeys exhibit similarities attributed to their shared geographic origin. In contrast, Ulmo honey was found to exhibit distinct characteristics, indicating a divergence in its geographical origin.

## 3. Challenges in Honey Phenolic Research

Recent studies have shown that variations in extraction methods can lead to significant variations in polyphenol profiles. For instance, a study by Michalkiewicz et al. [134] demonstrated that using different solvents fractions and sorbent materials could affect the selectivity and polyphenol content of different samples. This inconsistency demonstrates the need for a standardized extraction protocol that can be universally applied. Moreover, the process of honey extraction plays a crucial role in obtaining microheterogeneity in phenolic fractions from either liquid or solid matrices. To maintain stability and avoid fluctuations that could affect the co-elution of unwanted phenolic compounds, extraction factors such as the choice of solvent, and physico-chemical parameters, like time, temperature, pH, solvent-to-sample ratio, and extraction cycles, must be carefully selected [135,136].

In addition, the lack of harmonized protocols complicates the validation of polyphenol markers across different studies. For example, different sample preparation approaches, such as filtration, extraction, and dilution steps, can lead to different honey polyphenolic profiles [127]. Integrating polyphenol data with multivariate analysis techniques, such as principal component analysis (PCA) and partial least squares (PLS), have been successfully used to distinguish between different honey types based on their polyphenol content. These techniques have allowed for the identification of patterns and correlations that might not be evident through a univariate analysis [137].

## 4. Conclusions

In conclusion, the challenges faced by the U.S. honey market, particularly the issue of adulteration, highlight the need for effective tools to verify honey authenticity. Honey polyphenolic and sugar research holds promise as a crucial way to readily identify and authentic honey. It is our view that by studying these two constituents extensively and establishing the range of their amounts in honeys from different geographical regions, the ease of identifying adulterated honeys may be enhanced. However, this requires overcoming the challenges of inherent variability and method standardization. While most of the honey samples discussed in this review were within the *Codex Alimentarius* limits, a few were found to be below the acceptable range. So far, the sugar components of honeys produced in the United have not been extensively studied, emphasizing the need for further studies to explore reliable markers for honey authentication. By adopting standardized analytical approaches, integrating advanced techniques, and furthering research on U.S. honey varieties, the U.S. honey industry can better protect its products, ensure consumer trust, and maintain the high standards that genuine honey producers uphold.
